# P-1494. Risk of immune thrombocytopenia after second dose of a measles containing vaccine

**DOI:** 10.1093/ofid/ofaf695.1678

**Published:** 2026-01-11

**Authors:** Ousseny Zerbo, Sharareh Modaressi, Bruce Fireman, Pat Ross, Kristin Goddard, Jennifer Nelson, Jason Glanz, Stephanie Irving, Bruno Lewin, Malini B DeSilva, Joshua T B Williams, Maria Sundaram, Nicola P Klein

**Affiliations:** Division of Research Kaiser Permanente Vaccine Study Center, Oakland, CA; Kaiser Permanente Vaccine Study Center, Oakland, California; Division of Research Kaiser Permanente Vaccine Study Center, Oakland, CA; Kaiser Permanente Vaccine Study Center, Oakland, California; Division of Research Kaiser Permanente Vaccine Study Center, Oakland, CA; Kaiser Permanente Washington Health Research Institute, Seattle, Washington; Institute for Health Research, Kaiser Permanente Colorado, Denver, Colorado, Denver, Colorado; Kaiser Permanente Center for Health Research, Portland, Oregon; Kaiser Permanente Department of Research and Evaluation, Pasadena, CA; HealthPartners Institute, Bloomington, Minnesota; Denver Health, Denver, Colorado; Marshfield Clinic Research Institute, Marshfield, Wisconsin; Division of Research Kaiser Permanente Vaccine Study Center, Oakland, CA

## Abstract

**Background:**

Studies reported elevated risk of immune thrombocytopenia (ITP) after the first dose of a measles-containing vaccine (MCV), including combined measles, mumps, and rubella vaccines, with varicella (MMRV) or without (MMR). Whether risk is also elevated after the second MCV dose is not fully understood. The objective of the study was to evaluate whether MCV administered between ages 4-6 years (typically second dose) is associated with increased risk of ITP.

**Methods:**

We conducted a retrospective study from 2000 through 2022 at 8 integrated healthcare systems within the Vaccine Safety Datalink. We included children who received their MCV between ages 4–6 years and were members of their health system at least 180 days prior to vaccination through 181 days after vaccination. Potential ITP cases were identified using International Classification of Diseases (ICD) 9^th^ and 10^th^ Revision codes 287.31 and D69.3 and presence of platelet count < 100,000/µL. These potential cases were then confirmed by medical record review.

We compared the risk of ITP during days 15–28 (primary risk interval) and 1–42 (secondary risk interval) after vaccination with the risk during days 57–181 after vaccination (comparison interval). We used an exact binomial model to estimate relative risks and 95% confidence intervals (CI). Analyses were conducted for any MCV and separately by MMRV and MMR.

**Results:**

Among 60 chart-confirmed ITP cases, 4 were confirmed during the 15–28-day risk interval, 19 were confirmed during the 1–42-day risk interval and 41 during the 57–181-day comparison interval. Compared with the comparison interval, the relative risk (RR) of ITP following any MCV was 0.93 (95% CI 0.27–2.56) during the 15–28-day risk interval and 1.16 (95% CI:0.57–2.24) during the 1–42-day risk interval. Compared with the comparison interval, the RR of ITP during the 15–28-day risk interval was 1.53 (95% CI 0.34–5.04) after MMRV and 0.43 (95% CI 0.02–2.74) after MMR. The RR of ITP during the 1–42-day risk interval was 1.44 (95% CI 0.58–3.33) after MMRV and 0.82 (95% CI 0.25–2.42) after MMR. The numbers were too small to compare RR by MCV type for the 15-28-day risk interval.

**Conclusion:**

Among children aged 4–6 years, MCVs were not associated with increased risk of ITP. Risk did not vary by MCV type.

**Disclosures:**

Ousseny Zerbo, PhD, Centers for Disease Control and Prevention: Grant/Research Support|Moderna: Grant/Research Support|National Institutes of Health: Grant/Research Support|Pfizer: Grant/Research Support Stephanie Irving, MHS, Westat: Grant/Research Support Bruno Lewin, MD, Centers for Disease Control and Prevention: Grant/Research Support|National Institutes of Health: Grant/Research Support Malini B. DeSilva, MD, MPH, Centers for Disease Control and Prevention Vaccine Safety Datalink: Grant/Research Support|Westat: Grant/Research Support Maria Sundaram, PhD, MSPH, GSK: Grant/Research Support Nicola P. Klein, MD, PhD, AstraZeneca: Grant/Research Support|Centers for Disease Control and Prevention: Grant/Research Support|GlaxoSmithKline: Grant/Research Support|Janssen: Grant/Research Support|Merck: Grant/Research Support|Moderna: Grant/Research Support|Pfizer: Grant/Research Support|Sanofi Pasteur: Grant/Research Support|Seqirus: Grant/Research Support

